# Astrocytic GABAergic signaling in perioperative neurocognitive disorders: mechanisms and intervention strategies

**DOI:** 10.3389/fnins.2026.1990219

**Published:** 2026-09-28

**Authors:** Ting Tang, Xingcen Wang, HuiJuan Huang, JianZhi Shi, Hua Zhang

**Affiliations:** Department of Anesthesiology and Pain Management, Hangzhou Third People's Hospital, Hangzhou, China

**Keywords:** astrocyte, extrasynaptic GABA(A) receptor, GABA, general anesthesia, neuroinflammation, perioperative neurocognitive disorders, tonic inhibition, α5 subunit

## Abstract

Perioperative neurocognitive disorders (PND) are a major burden for older surgical patients, yet little is known about the cellular events that set them in motion, and no therapy currently changes their course. Anesthesia and amnesia after etomidate, sevoflurane, or isoflurane depend mainly on gamma-aminobutyric acid type A receptors (GABA(A)Rs), and an increasing body of work shows that astrocytes also respond to these drugs. This review examines an astrocyte-centered GABAergic cascade that may link anesthetic exposure to PND and grades the strength of the evidence at each node. When anesthetics engage astrocytic GABA(A)Rs, reactive astrogliosis follows, astrocytic GABA synthesis rises through the monoamine oxidase B (MAOB)-dependent putrescine route, and Best-1-mediated release along with weaker GABA transporter-3 (GAT-3) clearance lets ambient GABA accumulate. Extrasynaptic α5-GABA(A)Rs then become overactive, tonic inhibition of hippocampal and subcortical neurons may grow, synaptic plasticity falters, and cognition declines. We weigh the evidence behind each stage, distinguish mechanisms established in PND models from those extrapolated from other conditions, identify the astrocyte-specific experiments that remain to be performed, and consider interventions that could break the cascade, spanning α5-GABA(A)R negative modulation, MAOB blockade, GABA clearance repair, and dexmedetomidine. The astrocytic GABAergic axis therefore offers a promising candidate target for PND prevention and treatment, and we close by identifying priorities for translational research.

## Graphical Abstract

**Figure:**
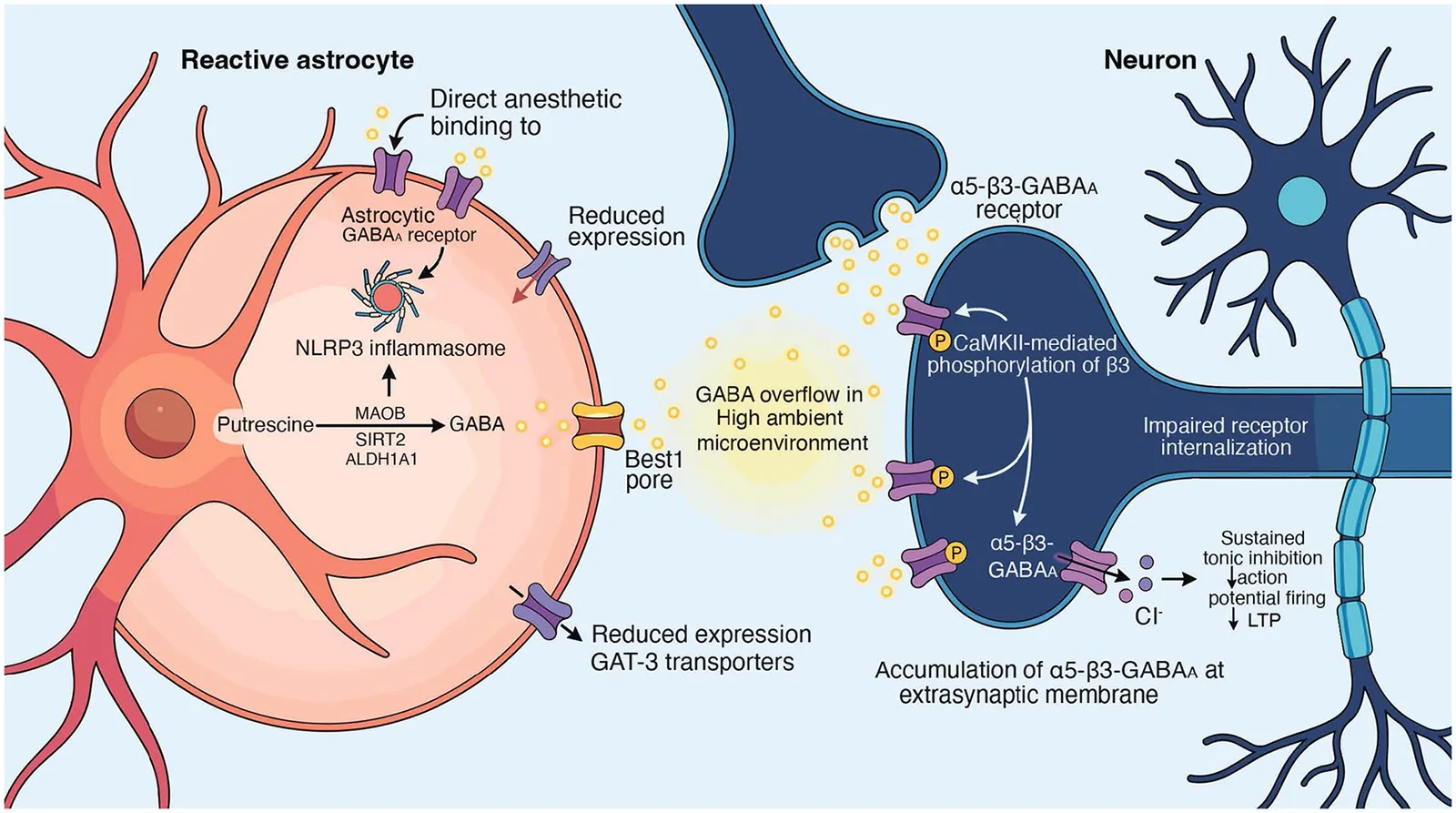
Molecular mechanisms of glial-neuronal GABAergic crosstalk driving tonic hyper-inhibition and synaptic failure in PND. General anesthetics act on astrocytic GABAA receptors and activate the NLRP3 inflammasome, which drives the non-canonical GABA biosynthetic machinery (MAOB, SIRT2, and ALDH1A1) to convert putrescine into excess intracellular GABA. GABA leaves the astrocyte through Best-1 anion channels, while downregulation of GAT-3 reuptake transporters impairs clearance, so ambient GABA accumulates in the microenvironment. On postsynaptic hippocampal pyramidal neurons, the volatile agent sevoflurane activates CaMKII, which phosphorylates the β3 subunit of extrasynaptic α5β3-GABAA receptors. Phosphorylation blocks clathrin-dependent endocytosis, causing receptors to accumulate on the extrasynaptic membrane. Persistent receptor accumulation together with sustained ambient GABA produces a non-desensitizing tonic inhibitory chloride current (Cl– influx). This hyperpolarizing shunt lowers neuronal firing, suppresses long-term potentiation, and shifts the circuit excitation/inhibition balance, providing a cellular substrate for perioperative neurocognitive disorders (PND).

## 1 Introduction

### 1.1 Perioperative neurocognitive disorders: a clinical problem in search of mechanisms

The term PND covers several cognitive problems that surface around surgery. These span impairment present before the operation, delirium that develops before or shortly afterward, delayed neurocognitive recovery, and the fully established postoperative disorder (Evered et al., 2022, 2018; Kong et al., 2022). In 2018, the earlier term Postoperative Cognitive Dysfunction (POCD) was replaced by this wider terminology. The change reflected the varied presentation of perioperative cognitive change and its frequent overlap with preexisting vulnerability (Evered et al., 2018). Delirium and longer-lasting cognitive decline remain common in elderly surgical populations. Up to 40% of patients show cognitive deterioration in the first postoperative week, and roughly 17% still do at 3 months (Zhao et al., 2024). Those outcomes predict longer hospitalization, loss of independence, and higher mortality (Anand et al., 2024; Sun et al., 2024). PND has also been associated with greater long-term dementia risk, Alzheimer disease included, though causality remains unsettled (Eckenhoff et al., 2020; Sun et al., 2024; Zhao et al., 2024).

PND has no single cause. Age-related brain vulnerability, surgical trauma, anesthetic exposure, perioperative inflammation, blood-brain barrier damage, and weak glymphatic clearance all contribute (Cheng et al., 2022; Dilmen et al., 2024; Eckenhoff et al., 2020; Zhao et al., 2024). Delirium appears in a considerable fraction of older patients after surgery, adds to their cognitive and physical burden (Rengel et al., 2018; Whitlock et al., 2011), and follows the same risk structure and pathological routes as delayed cognitive recovery (Dilmen et al., 2024; Kong et al., 2022). POCD is still encountered often after major operations, hip arthroplasty included (Xiao et al., 2020), and the processes behind it in older adults have drawn repeated review (Lin et al., 2020). Neuroinflammation, driven by activated microglia and astrocytes releasing pro-inflammatory cytokines, remains the most thoroughly studied pathway (Chen et al., 2025; Peng et al., 2023). Despite this work, no anesthetic or medication has demonstrably reduced PND frequency (Anand et al., 2024), and available prevention remains narrow, a gap that hints at unknown or poorly connected triggers (Calderon and Hemmings, 2026). Changes in gut microbiota now offer a peripheral explanation, since dysbiosis can propagate systemic and central inflammation through the gut-brain axis during PND (Zhou et al., 2025). Animal experiments further indicate that preoperative blood-brain barrier condition governs how much the aging brain suffers from anesthesia and surgery (Cao et al., 2025).

### 1.2 Anesthetics initiate the cascade: the GABA(A) receptor hypothesis

Sedation, hypnosis, and amnesia from etomidate, propofol, sevoflurane, and isoflurane rest chiefly on potentiation of GABA(A)Rs (Antkowiak and Rammes, 2019; Sigel and Steinmann, 2012). Memory loss is a hallmark of anesthesia, which gives these drug actions direct relevance to PND. For years, the working assumption was that GABA(A)R signaling returned to baseline after drug elimination (Zurek et al., 2014). Experimental findings overturned that assumption. A single *in vivo* etomidate dose increased tonic currents from α5 subunit-containing GABA(A)Rs [α5GABA(A)Rs]. Surface expression stayed elevated for at least a week, and isoflurane produced the same persistent shift. Memory and plasticity suffered under this sustained inhibition, and α5GABA(A)R blockade corrected both (Zurek et al., 2014). In aged mice, sevoflurane later proved equally disruptive, with CaMKII-mediated β3 phosphorylation preventing α5β3-GABA(A)R internalization and leaving receptors stranded on the neuronal surface (Wan et al., 2026). The neuronal GABAergic system therefore does not simply return to its pre-anesthetic state, and persistent tonic inhibition now stands as a plausible driver of anesthetic-linked cognitive loss (Orser, 2024; Orser et al., 2026; Wan et al., 2026; Zurek et al., 2014). Suspicion about anesthetic neurotoxicity in developing and aging brains has strengthened over the past 20 years, yet the clinical significance of postoperative cognitive change remains contested (Brambrink et al., 2012).

### 1.3 Astrocytes: from support cells to GABAergic signaling hubs

Astrocytes outnumber other glia in the central nervous system and are now viewed as genuine participants in synaptic transmission, metabolic support, and cognition (Khakh and Deneen, 2019; Meron Asher and Goshen, 2025; Nagai et al., 2021; Santello et al., 2019; Won et al., 2025). Their fine processes contact synapses and help shape information processing and behavior (Saint-Martin and Goda, 2023). Inflammatory or injurious signals drive these cells into a reactive phenotype marked by stereotyped morphological and transcriptional changes (Patani et al., 2023; Won et al., 2025). Work over recent years shows that reactive astrocytes synthesize and emit GABA, a bona fide gliotransmitter that holds tonic inhibition over nearby neurons (Cheong and Lee, 2025; Jo et al., 2014; Woo et al., 2018; Yoon and Lee, 2014). This GABAergic pathway first proved important for memory in Alzheimer disease models, where stopping astrocytic GABA production or release returned synaptic plasticity and learning to normal (Jo et al., 2014).

Earlier reviews have mapped how astrocytes take part in PND, from neuroinflammation and neurotransmitter balance to synaptic plasticity and neurotrophic support (Cao et al., 2022; He et al., 2024; Yang et al., 2024). What the field still lacks is a coherent scheme that anchors astrocytic GABAergic signaling in the anesthetic-to-PND sequence. Preclinical data now fill in every stage of the scheme. Anesthetics bind astrocytic GABA(A)Rs (Chung et al., 2021). Anesthesia and surgery turn astrocytes reactive and drive GABA overproduction (Cho et al., 2021; Li et al., 2020; Wang L. Y. et al., 2023). Astrocytic GABA strengthens tonic inhibition (Park et al., 2025; Tong et al., 2024). Excessive inhibition then damages plasticity and memory (Ge and Craig, 2024; Möhler and Rudolph, 2017; Zurek et al., 2014).

### 1.4 Scope and organization of this review

This review follows the chain from anesthetics (etomidate, sevoflurane, isoflurane) to astrocytic GABA(A) receptors, then to abnormal astrocytic GABA signaling, stronger neuronal tonic inhibition, and finally PND. We first describe the molecular machinery that lets reactive astrocytes synthesize, release, and clear GABA. We next ask how general anesthetics engage astrocytic and neuronal GABAergic targets. We then assess how strongly the evidence supports each link between astrocyte-derived GABA, tonic inhibition, and cognitive deficits after anesthesia and surgery, using three evidence levels: mechanisms established in PND models with astrocyte-specific manipulation, mechanisms supported by PND models or by non-PND models with astrocyte-specific manipulation, and effects extrapolated from other systems or from correlative data. We close by reviewing interventions aimed at this cascade and by listing unresolved questions. Adult and aged rodent models of PND anchor our analysis, supported by human clinical data where available, and claims built on indirect or correlative findings are flagged. Figure 1 summarizes the chronology of discoveries that produced this astrocyte-centered framework.

**Figure 1:**
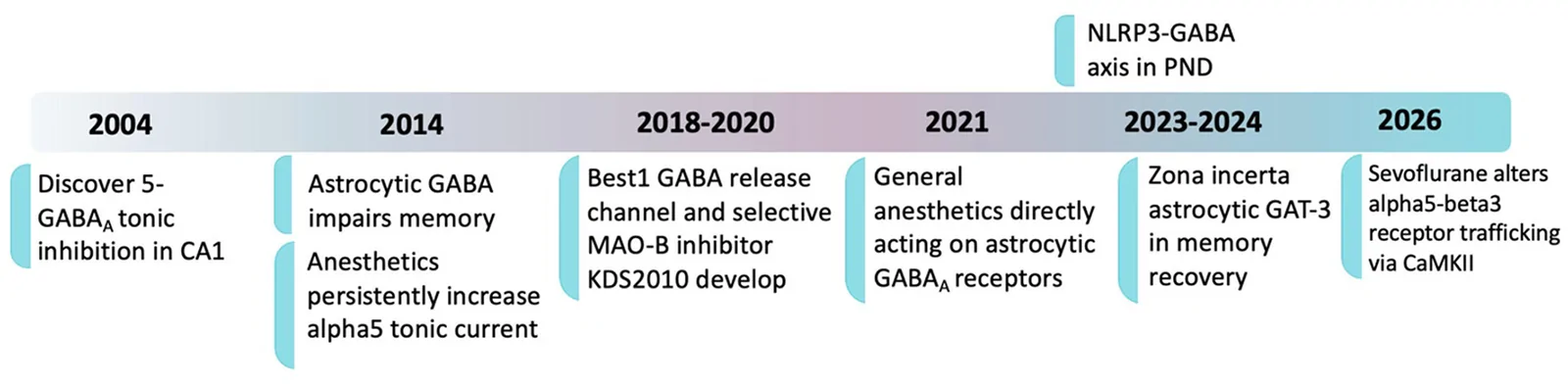
Chronological milestones in unraveling the astrocytic GABAergic axis in anesthetic-induced neurotoxicity and cognitive dysfunction. This timeline outlines key conceptual and mechanistic breakthroughs establishing the pivotal role of astrocytic GABA signaling and extrasynaptic tonic inhibition in anesthesia-induced cognitive impairment and PND. In 2004, extrasynaptic α5-subunit-containing GABAA receptors (α5-GABAARs) were first demonstrated to mediate tonic inhibitory currents in hippocampal CA1 pyramidal neurons. A decade later (2014), reactive astrocytic GABA release was recognized as an active gliotransmitter impairing memory in Alzheimer's models, and general anesthetics (etomidate, isoflurane) were shown to produce long-lasting enhancements of α5 tonic conductances persisting well beyond pharmacological clearance. From 2018 to 2020, the Best-1 anion channel was identified as the primary non-vesicular conduit for astrocytic GABA release, accompanied by the design of highly selective, reversible MAOB inhibitors (KDS2010) and proof that excess astrocytic GABA drives regional hypometabolism. Direct evidence that general anesthetics act on astrocytic GABAA receptors to orchestrate neuron-glia signaling emerged in 2021. Between 2023 and 2024, the astrocytic NLRP3 inflammasome–GABA axis was shown to drive surgery-induced cognitive impairment, and astrocytic GAT-3 downregulation in the zona incerta was identified as a critical clearance deficit causing tonic hyper-inhibition. By 2026, the molecular cascade was delineated, revealing that sevoflurane impairs α5β3-GABAAR trafficking and internalization via CaMKII-mediated phosphorylation of the β3 subunit, cementing the unified glial-neuronal framework of PND.

## 2 The astrocytic GABAergic machinery

### 2.1 GABA synthesis in reactive astrocytes: the MAOB-dependent putrescine pathway

Neurons form GABA from glutamate via glutamic acid decarboxylase (GAD). Reactive astrocytes rely instead on a GAD-independent route that converts putrescine to GABA and requires monoamine oxidase B (MAOB); quiescent astrocytes express this machinery at much lower levels (Jo et al., 2014; Woo et al., 2018; Yoon and Lee, 2014). In Alzheimer disease models, reactive astrocytes express high MAOB, build up GABA, and export it through bestrophin 1 (Best-1) channels. Blocking MAOB or deleting this synthetic route lowers astrocytic GABA and rescues memory (Jo et al., 2014). Steps downstream of MAOB are now mapped. Aldehyde dehydrogenase 1 family member A1 (ALDH1A1) and sirtuin 2 (SIRT2) catalyze successive reactions of the putrescine-to-GABA conversion, and astrocyte-selective SIRT2 silencing curbs astrocytic GABA production and partly improves memory in APP/PS1 mice (Bhalla et al., 2025). The pathway is conserved across regions and diseases. Astrocytic MAOB and GABA are elevated in the hippocampus of patients with Alzheimer disease 34 and in reactive astrocytes after cortical stroke, where tonic inhibition of neighboring neurons produces hypometabolism (Nam et al., 2020), and both features also appear in Parkinson disease and spinal cord injury models (Lee et al., 2025; Nam et al., 2022). Human post-mortem and bioinformatics work supports higher astrocytic MAOB and GABA-related enzyme expression in reactive astrocytes of Alzheimer disease brains (Assefa et al., 2018; Viejo et al., 2022).

Genetic studies show that astrocytic MAOB is required for tonic GABA signaling and sufficient to drive it. In cerebellar granule cells and striatal medium spiny neurons, astrocyte-specific MAOB knockout cuts tonic GABA currents by about 75% (Lee et al., 2022). Astrocyte-specific MAOB overexpression does the opposite, increasing tonic GABA release and lowering neuronal excitability and motor performance (Woo et al., 2018). In the mediodorsal thalamus, microglial signals act on astrocytes through a cytokine-dependent pathway involving Vaccinia-related kinase 2 (VRK2), Diamine oxidase (DAO), and ALDH1A1, leading to tonic GABA production. Thus, astrocytic GABA synthesis in this region is controlled both by microglial crosstalk and by the intrinsic reactivity of the astrocyte itself (Lee et al., 2026). GABA transaminase (GABA-T), the only enzyme that degrades GABA, sets astrocytic GABA content. Suppressing astrocytic GABA-T increases tonic GABA release, enhances tonic inhibition of dentate granule cells, and disrupts dorsal hippocampus-dependent spatial memory, all reversed by astrocyte-specific GABA-T rescue (Park et al., 2025). Together these data position the astrocytic GABA synthetic and catabolic machinery as a tunable control point for inhibitory tone. Table 1 shows preclinical evidence that links anesthetics to tonic inhibition and cognitive outcomes and indicates whether astrocytic involvement was assessed.

**Table 1:** Evidence linking anesthetics to tonic inhibition and cognitive outcomes, with astrocytic involvement indicated.

Model/agent	Astrocytic change	Neuronal/cognitive outcome	References
Etomidate or isoflurane, young adult mice	Not examined^*^	Persistent increase in tonic current and surface α5GABA(A)Rs for ≥1 week; impaired memory and LTP; reversed by α5 inhibition	Zurek et al., 2014
Sevoflurane, aged mice	Not examined^*^	CaMKII-dependent β3 phosphorylation impairs α5β3 subunit-containing GABA(A) receptor [α5β3GABA(A)R] internalization; cognitive impairment	Wan et al., 2026
Hippocampal slices, etomidate/sevoflurane	Increased astrocytic GABA(A)R charge transfer	Astrocyte-conditioned soluble factors increase neuronal GABA(A)R surface expression	Chung et al., 2021
Etomidate, 18-month-old mice	Microglia-dependent A1 astrocyte activation	Increased inhibitory synaptic transmission; cognitive impairment at 1-3 weeks	Li et al., 2020
Tibial fracture, aged mice	NLRP3-dependent excessive astrocytic GABA	Impulsive-like behavior and cognitive impairment; improved by selegiline or astrocytic NLRP3 knockout	Wang L. Y. et al., 2023
Laparotomy, mice	Astrocyte activation with increased MAOB and GABA	Postoperative pain and cognitive dysfunction; improved by CX3CR1/CX3CL1 blockade	Cho et al., 2021
Laparotomy, mice	Reactive astrocytes with GAT-3 downregulation in zona incerta	Increased tonic GABA currents in ZI neurons; recognition memory impairment; GAT-3 upregulation rescued	Tong et al., 2024
GABA-T knockdown, mice	Increased astrocytic tonic GABA release	Enhanced tonic inhibition of dentate granule cells; impaired spatial memory; rescued by astrocytic GABA-T rescue	Park et al., 2025
Astrocyte-specific MAOB cKO	Loss of astrocytic MAOB and GABA synthesis	~75% reduction of tonic GABA currents in granule cells and medium spiny neurons	Lee et al., 2022

^*^The first two studies are included because they define the neuronal α5GABA(A)R/tonic-inhibition endpoint of the proposed cascade; astrocytic involvement was not assessed in those studies.

### 2.2 GABA release from astrocytes: best-1 and beyond

Astrocytes export GABA through non-vesicular paths, an arrangement that suits the steady background levels these cells generate. Bestrophin 1 (Best-1), a calcium-gated anion channel, proved to carry GABA out of reactive and resting astrocytes (Jo et al., 2014; Woo et al., 2018). Genetic loss or pharmacological block of Best-1 lowers tonic GABA currents and heightens neuronal excitability, while added astrocytic GABA release does the reverse (Woo et al., 2018). The manipulations were performed in both resting and reactive astrocytes, and the effects were qualitatively similar. Other candidate routes include swelling-activated channels, exocytosis, and transporter reversal, and their contributions probably vary by brain region and disease context (Cheong and Lee, 2025; Yoon and Lee, 2014). What matters for the PND cascade is the tonic, largely action-potential-independent nature of this release, which keeps a constant supply of ambient GABA available to high-affinity extrasynaptic receptors (Park et al., 2025; Woo et al., 2018). This tonic release mode is present in both resting and reactive astrocytes, but its magnitude is substantially higher in the reactive state. Temporal control adds another dimension. Circadian GABA production by suprachiasmatic nucleus astrocytes synchronizes neuronal clocks (Ness et al., 2025). If anesthesia or surgery disturbs this daily rhythm, the sleep and circadian problems that accompany PND could follow.

### 2.3 GABA clearance: the role of astrocytic GABA transporters

Ambient GABA is shaped as much by reuptake as by release, and GABA transporters (GATs) handle that removal. Astrocytes rely on GAT-3, whereas neurons depend mostly on GABA transporter 1(GAT-1) (Sa et al., 2022; Tong et al., 2024). GAT-3 is expressed in both resting and reactive astrocytes, but its surface expression is downregulated during reactive transformation. Losing astrocytic GAT-3 has been connected to higher ambient GABA and stronger tonic inhibition in several conditions. In experimental autoimmune encephalomyelitis, reduced surface GAT-3 on hippocampal astrocytes coincides with more α5-GABA(A)R expression and greater CA1 tonic inhibition (Kammel et al., 2018). In a surgical PND model, ZI reactive astrocytes downregulate GAT-3, ZI neurons acquire larger tonic GABA currents, and GAT-3 restoration in these cells removes the surgery-linked recognition memory deficit (Tong et al., 2024). Circuit-level experiments strengthen this link, because optogenetic activation of the prelimbic cortex-to-ZI projection raises astrocytic GAT-3 and hastens recovery of postoperative recognition memory (Tong et al., 2026). Clearance itself sits under molecular control. STING regulates GAT-1 and GAT-3 transcription. When this regulation fails, ambient GABA climbs, tonic GABA(A)R inhibition of hippocampal principal neurons strengthens, and spatial learning plus working memory deteriorate (Neupane et al., 2024). Even outside disease models, acute inhibition of astrocytic GAT-3 in resting rat hippocampal astrocytes disrupts memory consolidation and retrieval through mechanisms tied to protein synthesis, so transporter-dependent clearance directly sustains hippocampal memory processes (Riboldi et al., 2026). GABA-T also helps control the releasable astrocytic GABA pool by degrading the transmitter inside the cell, acting in parallel with the transporters (Park et al., 2025).

### 2.4 Astrocytic GABA(A) receptors: an overlooked anesthetic target

GABA(A)Rs exist on astrocytes, but their normal duties remain largely unexplored. Astrocytic receptors differ from neuronal GABA(A)Rs in subunit composition and subcellular distribution, and they are positioned to link GABA sensing to intracellular calcium changes and downstream signaling. Their pharmacological profile, including sensitivity to neurosteroids and subunit-selective ligands, has been characterized mainly in reduced preparations of resting astrocytes (Chung et al., 2021). These receptors are present in resting astrocytes and are modulated further during reactive transformation, which makes them a plausible site of anesthetic action. The strength of the evidence for this node is graded in Table 2.

**Table 2:** Evidence grading for individual nodes of the astrocytic GABAergic cascade.

Cascade node	Model systems	Astrocyte-specific manipulation in PND	Evidence level
Anesthetic potentiation of astrocytic GABA(A)Rs	Hippocampal slices; astrocyte-neuron co-cultures	Not performed in PND models	B (Chung et al., 2021)
Reactive astrogliosis and NLRP3 activation after anesthesia/surgery	Aged-mouse PND models	Yes (astrocyte-specific NLRP3 knockout)	A (Li et al., 2020; Wang G. et al., 2023; Wang L. Y. et al., 2023)
MAOB-dependent GABA synthesis	Alzheimer disease mice; cerebellar and striatal circuits	Performed in non-PND models only	B (Cho et al., 2021; Jo et al., 2014; Lee et al., 2022; Park et al., 2019; Wang L. Y. et al., 2023; Woo et al., 2018)
ALDH1A1- and SIRT2-dependent GABA synthesis	Alzheimer disease models	Not performed in PND models	B (Bhalla et al., 2025)
Best-1-mediated GABA release	Alzheimer disease models; cerebellar circuits	Not performed in PND models	B (Jo et al., 2014; Woo et al., 2018)
GAT-3 downregulation and impaired GABA clearance	Surgical PND model (zona incerta); EAE	Yes (GAT-3 restoration in zona incerta)	A (Kammel et al., 2018; Tong et al., 2026, 2024)
Enhanced tonic inhibition	Etomidate and isoflurane models; zona incerta PND model	Partial (α5 modulation, not astrocyte-specific)	B (Li et al., 2020; Tong et al., 2024; Wan et al., 2026; Zurek et al., 2014)
Cognitive impairment and PND	Aged-mouse perioperative models	Full cascade not tested with astrocyte-specific tools	C (Cho et al., 2021; Li et al., 2020; Tong et al., 2024; Wan et al., 2026; Wang L. Y. et al., 2023; Zurek et al., 2014)

Level A, supported by PND models with astrocyte-specific manipulation; Level B, supported by PND models without astrocyte-specific manipulation or by non-PND models with astrocyte-specific manipulation; Level C, extrapolated from other systems, other cell types, or correlative evidence.

## 3 General anesthetics engage astrocytic and neuronal GABAergic targets

### 3.1 Anesthetic modulation of astrocytic GABA(A) receptors

Anesthetics act directly on astrocytic GABA(A)Rs. In hippocampal slices, focal GABA evoked picrotoxin-sensitive inward currents in stratum radiatum astrocytes, and etomidate (100 μM) and sevoflurane (532 μM) both increased charge transfer, with etomidate also increasing peak amplitude and both drugs prolonging current decay (Chung et al., 2021). Binding is only the first step; the functional consequence is a change in astrocytic signaling. Potentiation of these receptors raises intracellular calcium and promotes the release of gliotransmitters, and astrocytes exposed to anesthetics release soluble factors that increase the surface expression and function of neuronal GABA(A)Rs (Chung et al., 2021). Transcriptional responses and longer-lasting changes in astrocytic phenotype could follow, although direct evidence for such coupling in PND models is scarce. Whether astrocytic GABA(A)R activation is required for the sustained increase in tonic inhibition described after anesthesia remains untested (Chung et al., 2021; Orser, 2024), and current data favor parallel engagement of astrocytic and neuronal α5GABA(A)Rs.

### 3.2 Anesthesia and surgery drive reactive astrogliosis and astrocytic GABA synthesis

Anesthesia and surgical trauma share a neuroinflammatory program that pushes astrocytes into a reactive state. In an etomidate-based PND model, hippocampal microglia in aged mice activated within 1 week, with A1-reactive astrocytes following at 3 weeks. Microglial depletion before etomidate weakened the astrocytic response and preserved cognition, whereas microglial activation after etomidate worsened astrogliosis and cognitive loss (Li et al., 2020). Recordings in this model showed lasting increases in inhibitory synaptic transmission, suggesting the microglia-to-astrocyte cascade ultimately biases hippocampal circuits toward inhibition (Li et al., 2020). In aged mice subjected to tibial fracture, astrocyte-specific knockout of NOD-like receptor protein 3 (NLRP3) reduced excess GABA in reactive hippocampal astrocytes, blunted impulsive behavior and cognitive impairment, and restored neuronal integrity (Wang L. Y. et al., 2023). Blocking CX3CR1/CX3CL1 signaling after laparotomy similarly suppressed hippocampal astrocyte activation, MAOB expression, and GABA production while improving pain and cognitive outcomes (Cho et al., 2021). The inflammatory cytokine TL1A aggravated POCD through NLRP3-dependent A1 astrocyte differentiation. NLRP3 knockout or pharmacological inhibition blocked this action, placing the NLRP3-astrocyte axis at the core of anesthesia/surgery-induced cognitive injury (Wang G. et al., 2023).

Two astrocytic changes outside the GABA synthetic pathway nevertheless bear on inhibitory tone and are therefore retained in this section. Prolonged isoflurane exposure weakens connexin 43 (Cx43)-dependent gap junction coupling and increases hemichannel activity, and anesthesia and surgery promote astrocytic phagocytosis of hippocampal terminals in the medial prefrontal cortex, which impairs memory consolidation. The remaining evidence on astrocytic dysfunction beyond GABA synthesis is summarized in Section 4.5.

### 3.3 Neuronal α5GABA(A) receptors: persistent enhancement of tonic inhibition

On the neuronal side, the α5 subunit is the defining component of the GABA(A) receptors that generate hippocampal tonic inhibition, as shown by the loss of tonic current in α5-subunit knockout mice and by the selective pharmacology of α5-preferring compounds (Caraiscos et al., 2004; Mohamad and Has, 2019; Orser, 2024; Rudolph and Möhler, 2014; Sallard et al., 2021; Walker and Semyanov, 2008). Tonic inhibition was first described as a non-synaptic, persistent conductance of CA1 pyramidal neurons. It is carried mainly by α5GABA(A)Rs, which sense GABA with high affinity, desensitize slowly, and differ pharmacologically from synaptic receptors. These tonic conductances respond strongly to general anesthetics, sevoflurane and propofol included (Orser, 2024), and account for much of the network-synchronizing and amnestic action of GABAergic drugs (Sallard et al., 2021; Walker and Semyanov, 2008). The pharmacology of α5-containing receptors rests on a well-characterized structural basis (Scott and Aricescu, 2019; Sigel and Steinmann, 2012; Walker and Semyanov, 2008), and α5GABA(A)Rs now stand as bidirectional regulators of cognition (Mohamad and Has, 2019; Rudolph and Möhler, 2014).

That anesthetics leave a lasting mark on tonic inhibition is now well established. Zurek and colleagues showed that one sedative exposure to etomidate or isoflurane raises α5GABA(A)R-dependent tonic currents and receptor surface expression for up to a week, impairing hippocampal memory and long-term potentiation (LTP) (Zurek et al., 2014). Wan and colleagues extended this observation to sevoflurane in aged mice. CaMKII-dependent phosphorylation of the β3 subunit emerged as the trigger that blocks α5β3-GABA(A)R internalization and promotes receptor accumulation at the membrane (Wan et al., 2026). An accompanying synthesis proposes that three complementary routes combine to create excessive inhibition in PND. Neuronal GABA(A)R accumulation, aberrant astrocytic GABA release, and rising hydrogen peroxide production each contribute, and the first two converge on stronger tonic inhibitory conductance (Calderon and Hemmings, 2026; Orser et al., 2026). This view connects the astrocytic GABAergic machinery of Section 2 with the anesthetic actions discussed here.

## 4 Enhanced tonic inhibition: bridging astrocytic GABA to PND

### 4.1 Tonic inhibition, α5GABA(A) receptors, and memory

Tonic inhibition arises from extrasynaptic GABA(A)Rs whose high GABA affinity and slow desensitization let them respond to low ambient transmitter levels (Caraiscos et al., 2004; Walker and Semyanov, 2008). Most of this conductance in the hippocampus flows through α5GABA(A)Rs, which tune excitability, spike timing, and the excitation/inhibition balance behind network oscillations and plasticity (Caraiscos et al., 2004; Sallard et al., 2021; Walker and Semyanov, 2008). Rodent experiments reveal a two-way relationship between tonic inhibition and memory. Negative modulation of α5-containing receptors strengthens hippocampus-dependent learning, positive modulation weakens it, and α5GABA(A)Rs therefore act as a validated cognitive control point (Mohamad and Has, 2019; Möhler and Rudolph, 2017; Rudolph and Möhler, 2014). Genetic findings show that even small rises in tonic inhibition disrupt memory. Clptm1 acts as a chaperone that keeps GABA(A)R subunits in the endoplasmic reticulum. When only one allele remains, phasic and tonic inhibition both increase. Hippocampal LTP and contextual fear memory collapse under these conditions, and L-655,708, the α5 negative allosteric modulator, restores both (Ge and Craig, 2024). In aged rats, memory failure accompanies overactive CA3 networks. Yet α5 agonists, not inverse agonists, improve memory there, a twist that exposes the context dependence of α5 pharmacology (Koh, 2012). Fragile X syndrome shows how tonic-inhibition loss can drive hyperexcitability (Curia et al., 2009). Schizophrenia, by contrast, carries altered tonic-inhibition markers in the prefrontal cortex (Maldonado-Avilés et al., 2009). Endogenous neurosteroids add another control layer because they adjust GABA(A)R function, tonic conductances included, broadening the regulatory landscape of this signaling mode (Wang, 2011).

### 4.2 Astrocytic GABA as a source of pathological tonic inhibition

GABA from reactive astrocytes dictates tonic inhibitory tone in multiple disease models. In the Alzheimer disease dentate gyrus, astrocytic GABA engages presynaptic GABA(B) receptors and cuts granule cell spike probability. Halting astrocytic GABA synthesis or release restores plasticity and memory (Jo et al., 2014). Cerebellar GABA flowing through the MAOB-Best-1 route controls neuronal excitability, transmission, and motor output in both directions (Woo et al., 2018). After cortical stroke, MAOB-dependent astrocytic GABA suppresses glucose use by tonically inhibiting neighboring neurons, and MAOB blockade reverses the metabolic and behavioral changes (Nam et al., 2022). In the mediodorsal thalamus, microglial VRK2 feeds a cytokine-dependent DAO-ALDH1A1 pathway that generates tonic GABA in astrocytes, and removing VRK2 shrinks thalamic tonic currents (Lee et al., 2026). Motor signs in Parkinson disease models arise when astrocytic GABA tonically inhibits dopaminergic neurons (Heo et al., 2020). Pain and mood circuits are also within the pathological reach of this transmitter. In the spinal dorsal horn, astrocytic GABA works with downregulation of the K+/Cl- cotransporter KCC2 to produce tonic excitation and neuropathic pain (Ju et al., 2024). This overlap matters because pain and PND frequently coexist after surgery. In a rat depression model, blocking astrocytic GABA restores prefrontal cortical synaptic plasticity (Srivastava et al., 2020). A general principle follows. Astrocytic GABA release regulates ambient GABA and tonic inhibition locally, and excess release alone can disrupt circuit activity and behavior.

### 4.3 Astrocytic GABA and tonic inhibition in PND models

Direct evidence now ties this astrocytic GABAergic axis to cognitive impairment induced by anesthesia and surgery. Laparotomy in the zona incerta (ZI), a subcortical node tied to attention and memory, triggered reactive astrogliosis, astrocytic GAT-3 downregulation, and larger tonic GABA currents in ZI neurons. Recognition memory was impaired in the same animals, and restoring astrocytic GAT-3 corrected both the electrophysiological and behavioral abnormalities (Tong et al., 2024). Circuit-level activation of the prelimbic cortex-to-ZI pathway raised astrocytic GAT-3 and sped recovery from surgery-induced recognition memory dysfunction, marking transporter restoration as a druggable node 59. In aged mice recovering from tibial fracture, the NLRP3-GABA axis in reactive hippocampal astrocytes drove impulsive behavior and cognitive impairment. Selegiline lowered excessive astrocytic GABA and eased the behavioral phenotype (Wang L. Y. et al., 2023). In the etomidate model, the delayed A1-astrocyte response tracked long-lasting increases in inhibitory transmission and cognitive deficits (Li et al., 2020). CX3CR1 blockade after laparotomy lowered astrocytic MAOB and GABA, and outcomes improved (Cho et al., 2021).

Together these observations support a working model in which anesthesia and surgery raise ambient GABA through two complementary routes. One route is direct anesthetic potentiation of astrocytic GABA(A)Rs and neuronal α5GABA(A)Rs (Chung et al., 2021; Zurek et al., 2014). The other route involves reactive-astrocyte-driven increases in MAOB-dependent GABA synthesis and release combined with reduced GAT-3-mediated clearance (Cho et al., 2021; Tong et al., 2024; Wang L. Y. et al., 2023). Stronger tonic inhibition then suppresses excitability and plasticity in hippocampus-dependent memory circuits (Ge and Craig, 2024; Möhler and Rudolph, 2017; Zurek et al., 2014). Additional astrocytic failures compound the network dysfunction (Figure 2), among them disrupted gap junction coupling (Dong et al., 2022), impaired metabolic support (Femenía et al., 2018; Li et al., 2025), and aberrant glutamate handling (Li et al., 2024). Single-nucleus transcriptomic profiling of hippocampal cells from aged mice supports this model. It uncovered PND-associated astrocytic states and connected excitation-inhibition imbalance, with disturbed inhibitory control of excitatory plasticity, to postoperative cognitive impairment (Wang et al., 2026).

**Figure 2:**
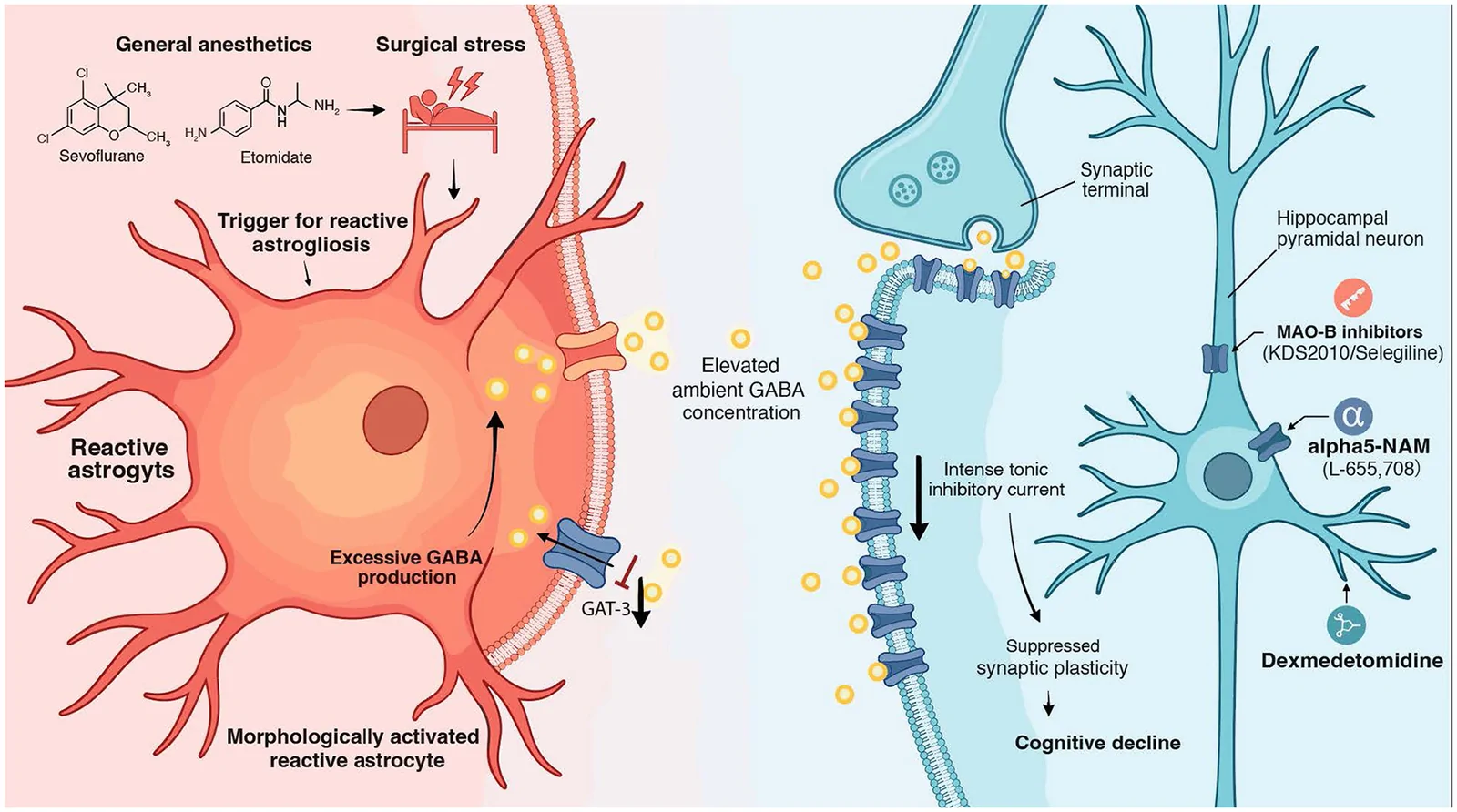
Circuit-level cascade linking anesthetic/surgical stress to perioperative neurocognitive disorders (PND) and therapeutic intervention points. General anesthetics (sevoflurane, etomidate) and surgical stress trigger reactive astrogliosis, leading to excessive GABA production and impaired GABA clearance in reactive astrocytes. The resulting elevation of ambient GABA overactivates extrasynaptic α5-containing GABA**(A)** receptors on hippocampal pyramidal neurons, producing an intense tonic inhibitory current. This sustained inhibition suppresses synaptic plasticity and ultimately leads to cognitive decline. The cascade can be interrupted at three therapeutic checkpoints: MAO-B inhibitors (KDS2010 or selegiline) reduce astrocytic GABA synthesis; α5-GABA**(A)**R negative allosteric modulators (L-655,708) attenuate tonic hyper-inhibition; and dexmedetomidine provides upstream neuroprotection by reducing neuroinflammation and circuit dysfunction. Molecular details of the underlying signaling machinery are shown in the Graphical Abstract.

### 4.4 Caveats and unresolved issues

Before this model is accepted wholesale, several caveats deserve attention. Few PND studies have managed direct tonic-inhibition recordings. The clearest evidence lies in the zona incerta and the etomidate model, where inhibitory transmission grew (Li et al., 2020; Tong et al., 2024). No hippocampal tonic-current recordings after anesthesia in aged animals exist yet. How much GABA leaves astrocytes varies across regions and models. In diet-induced obesity, MAOB and GABA rose in the arcuate nucleus, but enhanced neuronal GAT-1 uptake compensated and tonic inhibition stayed unchanged (Sa et al., 2022). Transporter status can thus hide or exaggerate changes in synthesis. How much of the cognitive injury traces to astrocytes vs. neurons is unresolved. Only astrocyte-specific manipulation of GABA synthesis or release in PND models, not systemic MAOB inhibition, can answer that question (Lee et al., 2022; Park et al., 2025). Age introduces further uncertainty. Because most mechanistic work used young adult mice (Jo et al., 2014; Zurek et al., 2014), how aging changes the picture remains open, given that PND strikes aged brains with distinct GABAergic tone and astrocytic behavior (Koh, 2012; McQuail et al., 2015; Tran et al., 2018; Zhao et al., 2024). Mitochondrial dysfunction compounds PND pathology (Liu et al., 2025), and peripheral metabolic disturbances that include omega-6 fatty acid dysregulation add to it (Chen et al., 2026), with both able to interact with the astrocytic GABAergic axis. Alternative explanations also deserve weight. Neuronal mechanisms, including persistent changes in α5GABA(A)R trafficking, and systemic inflammatory pathways that act independently of astrocytes could account for part of the cognitive phenotype. Several nodes of the proposed cascade rest on evidence from Alzheimer disease, stroke, experimental autoimmune encephalomyelitis, or cerebellar circuits, and their transfer to PND remains to be demonstrated with astrocyte-specific tools in perioperative models. Table 2 summarizes the evidence level assigned to each node.

Second, a critical distinction between correlative and causal evidence must be acknowledged. Pharmacological MAOB inhibition with selegiline attenuates excessive astrocytic GABA and improves behavioral outcomes in PND models. Astrocyte-specific NLRP3 knockout reverses surgery-induced cognitive impairment. These observations do not establish that astrocyte-derived GABA is necessary for anesthetic-induced cognitive impairment. The strongest causal evidence for the astrocytic MAOB-GABA axis originates from Alzheimer disease models and cerebellar motor function studies, in which astrocyte-specific MAOB conditional knockout reduces tonic GABA currents by approximately 75%. In PND models, astrocyte-specific genetic manipulation of GABA synthesis (MAOB, ALDH1A1, SIRT2), release (Best-1), or clearance (GAT-3) has not been systematically performed. Systemic MAOB inhibition cannot distinguish astrocytic from neuronal contributions, nor GABA-dependent from GABA-independent drug effects. This gap in causal evidence should temper interpretations of the cascade as a linear, unidirectional pathway from anesthetics to PND.

### 4.5 Complementary astrocytic mechanisms beyond GABA synthesis

Astrocytic changes beyond GABA synthesis also influence brain function after anesthesia and surgery. Prolonged isoflurane exposure weakens connexin 43 (Cx43)-dependent gap junction coupling between hippocampal astrocytes and increases hemichannel activity (Dong et al., 2022), and loss of hippocampal Cx43 after surgery in aged mice thins perineuronal nets and contributes to PND (Zhang Q. et al., 2025). Single-cell transcriptomic profiling has begun to resolve the heterogeneity of the reactive astrocyte response (Zhao et al., 2025). Macrophage migration inhibitory factor and endoplasmic reticulum stress-driven autophagy amplify this reactive program (Zeng et al., 2025; Zhang et al., 2023), whereas falling β-arrestin1 levels in postoperative delirium models worsen cognitive and inflammatory outcomes through Drp1-dependent mitochondrial fission (Hua et al., 2023). Laparotomy with sevoflurane also promotes tau phosphorylation and neuroinflammation (Yu et al., 2021), and anesthesia and surgery can trigger astrocytic phagocytosis of hippocampal terminals in the medial prefrontal cortex, impairing memory consolidation (Ma et al., 2024). Peripheral surgery further disturbs neuroglial metabolic coupling, with early hippocampal IL-6 elevation, altered astrocytic proteins for energy supply and glutamate recycling, reduced astrocytic calcium signaling, and lower lactate availability (Femenía et al., 2018). Chemogenetic activation of astrocytic Gq signaling in CA1, which raises astrocytic calcium, reduces anesthesia/surgery-induced cognitive and plasticity deficits in aged mice, supporting a causal role for astrocytic dysfunction rather than reactivity alone (Wang X. et al., 2022). Reviews of anesthetic neurotoxicity likewise identify astrocytes as direct targets whose impaired function contributes to POCD (Cao et al., 2022; He et al., 2024; Yang et al., 2024).

## 5 Intervention strategies targeting the astrocytic GABAergic cascade

### 5.1 Modulating α5GABA(A) receptors

Because enhanced tonic inhibition executes the cascade, α5GABA(A)R modulation offers the most direct intervention. Several nodes along this cascade have already passed preclinical or clinical validation, as Figure 3 and Table 3 outline. In the etomidate/isoflurane model, pharmacological blockade of α5GABA(A)Rs fully reversed anesthetic-induced memory deficits (Zurek et al., 2014). The negative allosteric modulator L-655,708 restores neuronal excitability and memory-related plasticity under pathological tonic inhibition, in experimental autoimmune encephalomyelitis (Kammel et al., 2018) and in Clptm1 haploinsufficiency (Ge and Craig, 2024). Partial inverse agonists at α5GABA(A)Rs have moved into clinical development as cognitive enhancers, which supports the translational promise of this strategy (Bhalla et al., 2025; Möhler and Rudolph, 2017). Yet aged rats with CA3 hyperactivity behaved in the opposite way, since α5 agonists, not inverse agonists, improved their memory (Curia et al., 2009). The direction of modulation must therefore fit the circuit state, and timing relative to anesthetic exposure needs careful study.

**Figure 3:**
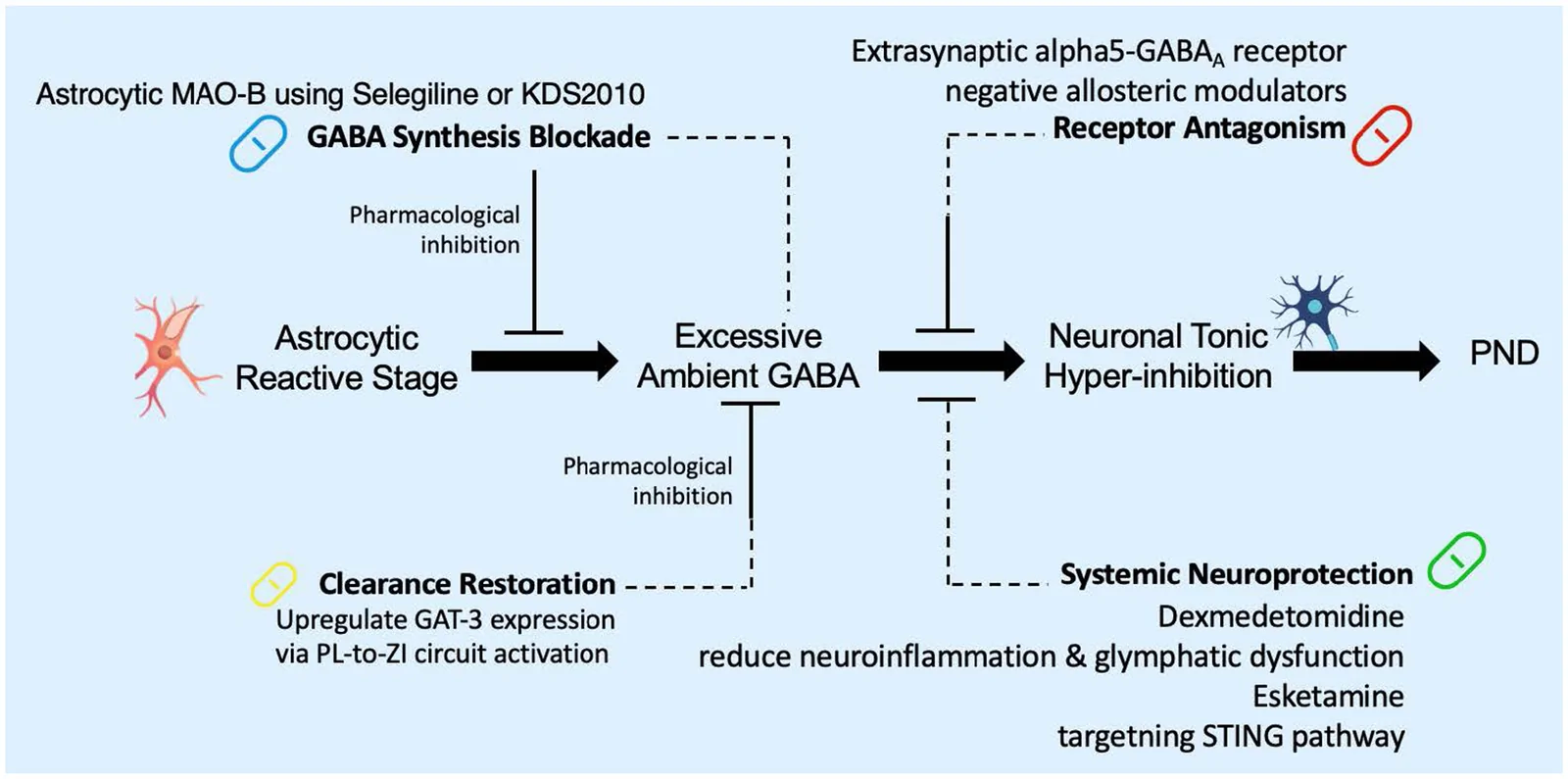
Multi-target translational intervention strategies interrupting the astrocytic GABAergic cascade in PND. A schematic roadmap delineating actionable therapeutic intervention nodes designed to disrupt the progression from anesthetic- and surgery-induced astrocytic reactivity to neuronal tonic hyper-inhibition and postoperative cognitive decline. The pathological continuum advances linearly from astrocytic reactivity, through excessive ambient GABA accumulation and neuronal hyper-inhibition, to overt PND. Four targeted intervention strategies are proposed to break this disease axis: (1) GABA Synthesis Blockade: Targeted inhibition of astrocytic monoamine oxidase B (MAOB) using clinical or selective inhibitors (selegiline, KDS2010) halts abnormal GABA generation through the putrescine cascade, reducing the total astrocytic GABA pool available for release. (2) Clearance Restoration: Restoring ambient GABA uptake through the upregulation of astrocytic GABA transporter-3 (GAT-3) expression—achieved pharmacologically or via circuit-level activation of the prelimbic cortex-to-zona incerta (PL-to-ZI) pathway, prevents transmitter overflow into the extracellular microenvironment. (3) Receptor Antagonism: Selective negative allosteric modulators (NAMs) directed against extrasynaptic α5-GABAA receptors (L-655,708) normalize heightened tonic inhibitory conductances without compromising essential synaptic GABAergic neurotransmission, thereby restoring hippocampal synaptic plasticity (LTP) and memory formation. (4) Systemic Neuroprotection: Clinically approved adjuvants provide upstream network protection; dexmedetomidine suppresses central neuroinflammation and alleviates glymphatic impairment, while esketamine mitigates glial pathology via the STING/TBK1 signaling pathway. Administered individually or in combination, these interventions effectively decouple anesthesia/surgery from downstream cognitive deterioration.

**Table 3:** Candidate interventions targeting the astrocytic GABAergic cascade in PND.

Target	Intervention	Model/evidence	Key outcome	Refs.
α5GABA(A)R	Negative allosteric modulation (L-655,708)	Etomidate/isoflurane model; EAE; Clptm1 haploinsufficiency	Reverses memory deficits; restores LTP and neuronal excitability	Ge and Craig, 2024; Kammel et al., 2018; Zurek et al., 2014
MAOB	Selegiline (irreversible)	Aged-mouse tibial fracture PND model	Reduces astrocytic GABA; improves impulsive-like behavior and cognition	Wang L. Y. et al., 2023
MAOB	KDS2010 (reversible, selective)	APP/PS1 AD model; stroke; spinal cord injury; phase 1 safety	Suppresses astrocytic GABA and astrogliosis; rescues memory; promotes repair	Lee et al., 2025; Nam et al., 2020; Park et al., 2019
Astrocytic GAT-3	Upregulation/circuit activation (PL-to-ZI)	Laparotomy PND model	Reduces tonic currents; restores recognition memory	Tong et al., 2026, 2024
Astrocytic GABA pool	GABA-T modulation (cell-specific)	Hippocampal GABA-T knockdown/rescue	Astrocyte-specific rescue normalizes tonic inhibition and spatial memory	Park et al., 2025
α2-adrenergic	Dexmedetomidine	RCT (*n* = 80); meta-analysis (59 RCTs, *n* = 7,713); cohort (*n* = 59,194)	Reduced delirium, emergence delirium, early POCD, and dementia risk	Karageorgos et al., 2026; Sun et al., 2025; Zhang et al., 2025b
STING/TBK1	Esketamine	Aged-rat laparotomy PND model	Alleviates postoperative cognitive decline; reduces astrocyte pathology	Li et al., 2022
Astrocytic Gq signaling	Chemogenetic activation (CNO)	Aged-mouse tibial fracture model	Attenuates cognitive impairment and synaptic plasticity deficits	Wang X. et al., 2022
Astrocytic glutamate/GLT-1	S-adenosylmethionine (SAMe)	Sevoflurane PND model (astrocytic FTO)	Normalizes m6A/GLT-1; improves cognitive performance	Li et al., 2024

### 5.2 Inhibiting astrocytic GABA synthesis: MAOB as a druggable node

Among all targets in the astrocytic GABA pathway, MAOB has the deepest validation. Selegiline, an irreversible MAOB inhibitor already in clinical use for Parkinson disease, trimmed excess GABA in reactive hippocampal astrocytes and lifted outcomes in an aged-mouse PND model (Wang L. Y. et al., 2023). Other perioperative models have also reported protective effects for the drug (Cho et al., 2021). Selegiline loses potency over time in Alzheimer disease models, because diamine oxidase rises to keep the GABA route alive. KDS2010, a highly selective reversible MAOB inhibitor (IC50 7.6 nM; 12,500-fold selectivity over MAO-A), solved that problem. It durably suppressed astrocytic GABA, strengthened synaptic transmission, and restored learning and memory in APP/PS1 mice (Park et al., 2019). The compound has shown benefit after stroke (Nam et al., 2020) and in spinal cord injury, where a phase 1 human safety profile proved favorable (Lee et al., 2025). Natural products expand the toolset. Suffruticosol B, a plant-derived MAOB inhibitor, lessens reactive astrogliosis and tonic GABA release and restores cognition in Alzheimer disease mice (Heo et al., 2025). MAOB inhibition also acts beyond transmitter synthesis, because it weakens reactive astrogliosis and scar formation after stab-wound injury (Chun et al., 2022), evidence of a wider anti-reactive profile. Whether these drugs protect against anesthesia and surgery is not yet known. Astrocyte-selective MAOB inhibition may outperform systemic blockade, and that possibility matters because deleting MAOB only in astrocytes already lowers tonic GABA currents (Lee et al., 2022).

### 5.3 Restoring GABA clearance and astrocytic GABA homeostasis

Restoring GABA clearance is now attracting attention as an intervention. Raising astrocytic GAT-3 in the zona incerta, directly or through prelimbic cortex-to-ZI circuit activation, reversed surgery-induced tonic inhibition and recognition memory deficits (Tong et al., 2026, 2024). Pharmacological control of the STING-GAT axis is possible because this pathway transcriptionally regulates GAT-1 and GAT-3. Ambient GABA and tonic inhibition can then be moved in either direction, yet the cognitive effect depends on the baseline state of the pathway (Neupane et al., 2024). Cell type complicates GABA-T targeting. Suppressing astrocytic GABA-T strengthens tonic inhibition and impairs spatial memory, whereas suppression in GABAergic neurons augments phasic inhibition, so any therapeutic gain would require cell-selective strategies (Park et al., 2025). Alongside the MAOB axis, these data map an astrocytic GABA metabolic cycle that spans synthesis, release, degradation, and reuptake, with a potentially druggable node at every stage.

### 5.4 Clinically available adjuncts acting upstream of the GABAergic cascade

Dexmedetomidine and esketamine are included here as clinically available adjuncts that act upstream of the astrocytic GABAergic cascade rather than as direct modulators of astrocytic GABA signaling. Dexmedetomidine, an α2-adrenergic agonist, has generated the largest body of PND intervention data. A double-blind trial gave older orthopedic patients dexmedetomidine sedation during regional anesthesia. Postoperative delirium fell to 4.8 from 38.4% with propofol, and emergence delirium and early POCD also declined (Karageorgos et al., 2026). Pooled data from 59 randomized trials and 7,713 orthopedic patients showed that dexmedetomidine lowered POCD (RR 0.59) and delirium (RR 0.49) (Zhang et al., 2025b). In 59,194 older hip fracture patients, postoperative use of the drug was tied to reduced dementia risk (adjusted HR 0.53) (Sun et al., 2025). Animal work credits dexmedetomidine with anti-inflammatory and microglial-quieting actions across several pathways (He et al., 2023; Wang et al., 2021). It also repairs glymphatic flow by restoring aquaporin-4 polarity and copying features of non-REM sleep (Zhang S. S. et al., 2025). Clinical guidance most often places this drug among PND prevention options (Kong et al., 2022). Elderly patients show less early POCD within 72 h after surgery in additional clinical reports (Wang W. et al., 2022). Meta-analyses further associate the drug with lower postoperative IL-6 (Lei et al., 2020). Current reviews stress its broader perioperative benefits beyond sedation (Zivadinovic et al., 2026). In aged mice, dexmedetomidine relieved POCD by repairing autophagy-lysosomal function, which held back NLRP3 inflammasome-mediated microglial activation and astrocytic A1 differentiation in the hippocampus (Wang Z. et al., 2025). Direct evidence that either drug modulates astrocytic GABA signaling is lacking, and whether part of their benefit reflects lower anesthetic requirements or reduced exposure of astrocytic and neuronal GABA(A)Rs remains to be tested.

Neuroinflammation and astrocytic reactivity are where several other agents join the cascade. Esketamine eased postoperative cognitive decline in aged rats through STING/TBK1 signaling and diminished astrocyte pathology (Li et al., 2022). In culture, ketamine stopped etomidate and sevoflurane from producing their lasting rise in GABA(A)R tonic currents in hippocampal neurons co-cultured with cortical astrocytes. That effect ran through BDNF-TrkB signaling rather than NMDA receptor blockade. Ketamine also spared mice from post-anesthetic recognition and spatial memory loss *in vivo* (Wang D-.S. et al., 2025). Ketamine is not without risk. Rodent studies connect the drug to glymphatic dysfunction and cognitive decline, so the perioperative benefit of ketamine-family agents will depend on dose and setting (Wu et al., 2024). Itaconate, a metabolite made endogenously, guarded cognition after anesthesia and surgery by turning on Nrf2 signaling, dampening microglial and astrocytic activation, and aiding neurogenesis (Kong et al., 2024). Microglial repopulation improved cognition after partial hepatectomy by cutting astrocytic CXCL1 and neutrophil entry through ZEB1/TGF-β1 signaling (Zhang et al., 2025a). Exosomes from astrocytes carrying miR-26a-5p improved neurocognitive recovery after long sevoflurane anesthesia via the NCAM/AKT/GSK3β/CRMP2 cascade (Li et al., 2024). Astrocytic Gq activation by chemogenetics in the hippocampus similarly protected aged mice in a surgical paradigm (Wang X. et al., 2022). Approaches that repair astrocytic glutamate handling, S-adenosylmethionine after sevoflurane-driven FTO-mediated GLT-1 loss among them, may work beside GABA-directed treatment (Li et al., 2026). Neutrophils have gained standing as a POCD target (Cheng et al., 2025), and irisin, a myokine released by exercise, reprograms microglia via STAT6 to avert surgery-induced cognitive dysfunction (Wang J. et al., 2025), adding to astrocyte-focused strategies.

### 5.5 Anesthetic strategy as a modifiable variable

If anesthetics set this astrocytic GABAergic cascade in motion, drug choice and dose double as prevention. The inhaled agents sevoflurane and isoflurane, together with intravenous etomidate, all potentiate tonic inhibition and astrocytic GABA(A)R activity (Chung et al., 2021; Wan et al., 2026; Zurek et al., 2014). Propofol potentiates tonic currents as well, but no data yet link it to lasting α5GABA(A)R upregulation (Orser, 2024). Randomized comparisons of intravenous vs. inhaled anesthesia in older patients are currently tracking blood-brain barrier integrity, neuroinflammation, delirium, and cognition (Wi et al., 2024). Guidance documents repeatedly identify deep anesthesia and benzodiazepines as alterable risk factors (Evered et al., 2018; Kong et al., 2022). Whether anesthetic depth alters the astrocytic GABA response, and whether regional or neuraxial techniques that cut GABAergic anesthetic exposure lower PND risk, are testable predictions of this framework (Anand et al., 2024; Wi et al., 2024).

## 6 Conclusions and future perspectives

The data reviewed here trace a continuous path that leads from anesthetic exposure, across astrocytic GABAergic signaling, to PND. Etomidate, sevoflurane, and isoflurane activate GABA(A)Rs located on both neurons and astrocytes (Chung et al., 2021; Zurek et al., 2014). Anesthesia and surgery shift astrocytes into reactivity, where MAOB-driven GABA synthesis, Best-1-mediated export, and weak GAT-3 uptake jointly raise ambient GABA (Cho et al., 2021; Li et al., 2020; Tong et al., 2024; Wang L. Y. et al., 2023). The surplus GABA drives extrasynaptic α5GABA(A)Rs beyond their normal range and deepens tonic inhibition (Caraiscos et al., 2004; Park et al., 2025; Tong et al., 2024). That added inhibition curbs synaptic plasticity and memory and leads to PND (Ge and Craig, 2024; Möhler and Rudolph, 2017; Zurek et al., 2014). The strength of the evidence differs across nodes: anesthetic effects on GABA(A)Rs and the downstream enhancement of tonic inhibition are supported by direct experiments, whereas astrocyte-specific manipulation of GABA synthesis, release, and clearance in PND models has not yet been performed. Hitting single nodes, from α5 negative modulation and MAOB blockade to GAT-3 restoration and dexmedetomidine, has yielded cognitive gains preclinically or in patients (Kammel et al., 2018; Karageorgos et al., 2026; Park et al., 2019; Tong et al., 2024; Wang L. Y. et al., 2023; Zurek et al., 2014). Taken together, the timeline in Figure 1, the mechanism in Figure 2, and the treatment map in Figure 3 portray PND as an astrocyte-centered disease with druggable checkpoints along a single mechanistic axis.

A small set of goals should organize future studies. Table 2 sets out the evidence level of each node and the astrocyte-specific experiments that are still required. First, tonic GABA currents need direct measurement in the hippocampus and cortex of aged mice after anesthesia that mirrors clinical practice. Such recordings would reveal whether zona incerta data transfer to the circuits that matter most for PND. Second, PND models need astrocyte-specific tools that alter GABA synthesis (MAOB, ALDH1A1, SIRT2), release (Best-1), and clearance (GAT-3). These experiments would establish whether astrocytic GABA is essential for anesthetic-induced cognitive loss (Lee et al., 2022; Park et al., 2025). Third, the interplay of anesthesia with aging requires attention, because aging reshapes astrocytic reactivity and GABAergic tone while most mechanistic data come from young animals (Curia et al., 2009; Orser et al., 2026; Zhao et al., 2024). Fourth, biomarkers tied to the astrocytic GABAergic axis, from MAOB activity to GABA-based imaging, might single out at-risk patients and gauge target engagement in clinical trials (Chen et al., 2025; Lee et al., 2025). Finally, α5-GABA(A)R negative modulators and MAOB inhibitors warrant prospective perioperative trials, using the safety and pharmacokinetic profiles already available for agents that include KDS2010 (Lee et al., 2025; Rudolph and Möhler, 2014). If astrocytic GABAergic signaling proves central, PND could stop being a syndrome without disease-modifying therapy and become one with several druggable targets arranged along one mechanistic axis (Calderon and Hemmings, 2026).
