# Supplementary material for: Astrocytic GABAergic signaling in perioperative neurocognitive disorders: mechanisms and intervention strategies
Source: Front Neurosci. 2026 Sep 28;20:1990219. doi: 10.3389/fnins.2026.1990219 (PMC13655799; doi:10.3389/fnins.2026.1990219)
Supplement: Supplementary file 1 [file Supplementary_file_1.docx]

**Supplement File
Search Strategy and Study Selection**

The narrative review synthesizes peer-reviewed literature on astrocytic GABAergic signaling in perioperative neurocognitive disorders (PND) (Cao et al., 2022; Kong et al., 2022; Zhou et al., 2025). We searched the Web of Science Core Collection from inception to 1 September 2026 using Boolean queries built from four keyword blocks covering the astrocyte population, PND-related cognitive terms, GABA/neuroinflammation-related mechanisms, and anesthesia/surgery (Table S1).

**Table S1.** Keyword blocks used in the Web of Science search strategy.

| **Block** | **Search terms** |
| --- | --- |
| #1 Astrocyte terms | TS=(astrocyte* OR astroglial*) |
| #2 Mechanism terms | TS=(GABA OR GABAergic OR glutamate OR "calcium signaling" OR connexin OR "gap junction" OR glymphatic OR AQP4 OR "microglia crosstalk" OR NLRP3 OR neuroinflammation) |
| #3 Cognition terms | TS=("cognitive dysfunction" OR POCD OR PND OR "perioperative neurocognitive") |
| #4 Perioperative terms | TS=(anesthesia OR anesthetic* OR surgery OR perioperative) |

The two retrieval queries were:

| Set #1 & #2 & #4 | TS=(astrocyte* OR astroglial*) AND TS=("cognitive dysfunction" OR POCD OR PND OR "perioperative neurocognitive") AND TS=(anesthesia OR anesthetic* OR surgery OR perioperative) |
| --- | --- |
| Set #1 & #2 & #3 | TS=(astrocyte* OR astroglial*) AND TS=("cognitive dysfunction" OR POCD OR PND OR "perioperative neurocognitive") AND TS=(GABA OR GABAergic OR glutamate OR "calcium signaling" OR connexin OR "gap junction" OR glymphatic OR AQP4 OR "microglia crosstalk" OR NLRP3 OR neuroinflammation) |

Results were restricted to English-language original articles and reviews with available abstracts and full text; preprints, conference abstracts, editorials, and corrections were excluded. Query Set #1 & #2 & #4 returned 231 records (214 in the Web of Science Core Collection, 209 after the language/article-type limits). Query Set #1 & #2 & #3 returned 1105 records, of which 917 were in the Web of Science Core Collection, 915 were English-language, and 896 met the article-type limit. Across the two queries, 1105 records were therefore retained after applying language and document-type limits, and removal of 141 duplicates left 964 unique Web of Science records. In parallel, 10 mechanism-specific PubMed/MEDLINE searches (astrocytic GABA machinery, tonic inhibition, anesthetic modulation of GABA(A) receptors, PND/POCD, dexmedetomidine, and related topics) identified 107 additional records.

| (astrocyte* OR "astroglial*")  AND ("anesthesia" OR "anesthetic*" OR "surgery" OR "perioperative")  AND ("postoperative cognitive dysfunction" OR "POCD" OR "PND" OR "perioperative neurocognitive") | 99 |
| --- | --- |
| ("astrocyte*" OR "astroglial*")  AND ("GABA" OR "GABAergic" OR "tonic inhibition" OR "GAT-3" OR "connexin" OR "gap junction" OR "AQP4")  AND ("postoperative cognitive dysfunction" OR "POCD") | 8 |
|  | 107 |

Two authors independently screened the titles and abstracts against predefined inclusion criteria (original research or reviews addressing astrocytic GABAergic signaling, tonic inhibition, anesthetic effects on GABA(A) receptors, or PND/POCD mechanisms and interventions), retaining 246 records for full-text assessment. After full-text evaluation, 132 records were excluded because they were off-topic mechanistic or cell-biology studies without perioperative context, duplicate or overlapping publications, or studies lacking extractable outcome data; 114 references were finally included in the review. Disagreements were resolved by discussion and consensus. The screening process is shown in Figure S1. Additional records were obtained by screening the reference lists of included articles, which allowed inclusion of seminal mechanistic studies that were not retrieved by the database queries; no journal-specific searches were performed.

**Figure S1.** Flowchart of the literature screening and filtering workflow derived from Web of Science and PubMed/MEDLINE

**References**

Cao, Y., Lin, X., Liu, X., Yu, K., Miao, H., & Li, T. (2022). The Role of Astrocytes in the Mechanism of Perioperative Neurocognitive Disorders. *Brain Sci*, *12*(11). <https://doi.org/10.3390/brainsci12111435>

Kong, H., Xu, L. M., & Wang, D. X. (2022). Perioperative neurocognitive disorders: A narrative review focusing on diagnosis, prevention, and treatment. *CNS Neurosci Ther*, *28*(8), 1147-1167. <https://doi.org/10.1111/cns.13873>

Zhou, Q., Chen, T., Wang, X., Xu, Z., Song, Y., Liu, S., Ge, Y., & Gao, J. (2025). Role of gut microbiota in neuroinflammation: a focus on perioperative neurocognitive disorders [Review]. *FRONTIERS IN CELLULAR AND INFECTION MICROBIOLOGY*, *15*, Article 1582909. <https://doi.org/10.3389/fcimb.2025.1582909>
